# Extra Supplement—The Nursing Mirror

**Published:** 1889-06-15

**Authors:** 


*The Hospital*, June 15, 1889. [*Extra Supplement*]

### The Nursing Mirror.

All Contributions for this Supplement should be addressed "The Nursing Mirror," care of the Editor, 139-140, Salisbury Court, Fleet Street, London, E.C.

### En Passant.

NURSES AS GUESTS.—On Wednesday, June 5, the nurses of the Torbay Hospital were kindly entertained at tea and supper by Miss Webb and Miss Black, of Meadwood Villa. These small outings are very pleasant and very good for nurses, especially during the summer months, when the heat is so oppressive within the hospital wards.

A JUBILEE CHAPLAIN.—The Queen has presented the Rev. A. L. B. Piele, vicar of Holy Trinity, Ventnor, and Honorary Chaplain to the Queen, to the Mastership and Chaplaincy of St. Katherine's Hospital, the Jubilee Nurses' Institution, founded in Regent's Park, London, with the surplus funds of the Women's National Tribute to Her Majesty on the occasion of her Jubilee.

PRINCESS LOUISE.—Owing to a shower of rain during the proceedings of opening the Children's Hospital at Leicester, the Princess Louise paid an unexpected visit to the wards of the infirmary. She spoke to several of the patients, and also to the nurse of St. Matthew's Ward, of whom she made enquiries as to the amount of study required in the nursing profession, and to whom she wished all success. Miss Rogers, the lady superintendent, went round with the Princess, to whom she had been presented by the Duke of Rutland at the commencement of the proceedings.

A NURSE COLLECTOR.—Saturday last was kept as Hospital Saturday at Richmond, and the usual processions and collections took place. On the railway bridge, Nurse Foley, of the Richmond Hospital, who will be remembered by many friends at The London, presided over one of the boxes. During the seven years Nurse Foley has been in charge of the Cambridge Ward, many of the watermen, not only of Richmond, but of the neighbourhood, have, in consequence of accidents, etc., been under her care, and it was partly at their request that she consented to give her services to the fund.

A VIGOROUS MATRON.—When the committee of Carmarthen Infirmary elected Miss Macintyre to her present post, they little knew what a perfervid Scot they were introducing into their midst. Miss Macintyre is slowly working a complete revolution in sleepy old Carmarthen, and her neighbours are delighted to help her. This lady with a garden is invited to send rhubarb, this carpenter is invited to make a foot-rest, and the children are asked to go and search the woods for flowers. Several members of the committee have been brought to see that among other things that demand urgent attention is the state of the ward floors, which are of deal, and from constant scrubbing and wear have become most uneven. The damp which necessarily evaporates from the inequalities in the board after scrubbing them, and the harbour which is given to dust and germs, is known to be so prejudicial to patients that it has been decided to be absolutely necessary either to relay the floors or replace them. The walls of the wards are to be repainted and colour washed, which will make the patients' surroundings much more cheerful than they are at present. The Carmarthen Infirmary will not know itself by the time Miss Macintyre has done with it, and the committee are to be congratulated on having so earnestly taken up this theme of improvements and alterations.

REGISTRATION OF MIDWIVES.—The Provisional Committee, to discuss the passing of a Bill for the registration of midwives, met on June 6, at Lady Fytzwygram's, 7, Grosvenor-crescent, Mrs. Henry Smith in the chair. This committee was the result of the meeting at Mrs. Rathbone's, 18, Prince's-gardens, May 23, and was very well attended. There was much business transacted, and the ladies present gave much practical help, and were unanimous in expressing their approval of the object of the meeting. The next committee to report progress will meet on June 25, at three o'clock in the afternoon, at 7, Grosvenor-crescent.

BANGOR INSTITUTION.—We have just received the annual report of the Bangor Institution of Trained Nurses for last year, which has been delayed in the printing. The institution is now ten years old, and reports steady growth. The nurse kept solely for attendance on the sick poor has so much to do that a second nurse is needed to aid her. Financially the institution did well last year, chiefly owing to the extra earnings of the nurse and the aid of the Bangor Choral Society, and also the Orchestral Society. After six years of constant service Miss Briggs, the lady superintendent, sent in her resignation, and the committee report the loss with regret.

SUMMER AFTERNOONS.—Just now, when fine weather is the rule, and daylight extends well into the evening, nurses ought to try and get a peep at the country, which is looking so fresh and beautiful. It is very easy to get away into green paths and shady woods from every part of London, and the slight effort necessary to make a start is more than repaid by the rest and refreshment got from the clear air and sunlight. Kew Gardens can be reached from any of the underground stations, the time from Victoria or Bishop's-road being about thirty minutes, the return fare, third class, about 1s. Of course, the palm-houses and fern-conservatories at Kew are very beautiful, but one of the prettiest and least-known parts of the gardens is the river bank, where wild flowers bloom and cockneys seldom come. An afternoon spent here with a friend, or a book, and then tea in the refreshment-room in the gardens (which is quite quiet and respectable), and then the return journey past the market gardens—now masses of bloom—makes a delightful and cheap outing for a summer afternoon. We hope if our space permits to suggest other little excursions to our readers as the weeks go by, for we feel that it is useless to recommend them concerts and lectures just now.

A CENSUS OF HOSPITAL WORKERS DURING THE YEAR 1888.—The following statement shows the numbers of the various officials engaged in the work of the Metropolitan Hospitals at the present time:

| | |
|---|---|
| Chaplains | 46 |
| Honorary Medical Officers | 1,260 |
| Paid Medical Officers | 301 |
| Secretaries, Clerks, and Collectors | 193 |
| Matrons | 102 |
| Nurses | 1,833 |
| Stewards and Housekeepers | 63 |
| Dispensers | 160 |
| Domestic Servants (Male) | 447 |
| " " (Female) | 1,062 |
| | 5,467 |

### Nurses and Registration.

*(From a Correspondent.)*

I was present at the meeting at Mrs. Jeune's house, in Wimpole-street, on the 5th; I listened to the papers of Mrs. Ormiston Chant and Miss Wood; I heard the questions of the one or two ladies who seemed to take more than a languid interest in the proceedings and the answers which were vouchsafed to them; and as I came away I could not but feel how very little had been said about the real meaning of this registration, which the meeting was supposed to advocate. There had been the usual, the classic, the fine old crusted allusions to Mesdames Gamp and Prigg, without which no meeting of the B. N. A. would be complete. Mrs. Ormiston Chant had argued that ladies might be brought to poverty, and illustrated the point by a story of a violin, a locket, and a pawnbroker, who defined a lady as a "high-stepper." She had also remarked in a casual way that nursing was a remunerative profession, and that, therefore, —I did not myself see where the *therefore* came in— registration was desirable. Miss Wood read letters from unhappy nurses who complained that unprofessional rivals were "taking the bread out of their mouths." Dr. Bedford Fenwick became lachrymose over the misdeeds of two *diplomées* of the Obstetrical Society, and longed for a register from which to erase their names. Princess Christian read over the resolutions in favour of obtaining a Royal charter and registration, and the meeting received them with perfect courtesy. And practically that was all. For the questions that were put were answered in a very unsatisfactory manner. One lady asked what the regulations of the desired charter would be, but Miss Wood would only say that three years' training would form an essential part of it, but for the rest, as it was not yet laid before the authorities, she could give no information. Another suggested the advisability of some satisfactory provision being made for nurses in their old age, such as is done in a nursing sisterhood she spoke of in Dijon, where a portion of the sisters' salary is reserved to form a pension. But, of course, no one hinted that such a thing as the National Pension Fund existed, and on a basis very similar to that spoken of. The "studied silence" which a certain periodical speaks of was certainly maintained for once. But the most awkward thing happened to Dr. Bedford Fenwick, when a lady asked a rather pertinent question as to the value of that medical register to which he had compared the nurses' register that is to be.

Dr. Bedford Fenwick had, as we have said, spoken in very touching terms of the need of a register. He had passed round a copy of the medical register among a company to whom it was simply a bulky red mystery. He had spoken of the time when the Council—by the way, who are to compose that council?—would strike a nurse's name off the register for unprofessional conduct,—we should like to know what would be defined as unprofessional conduct. He said that it would be easy, in the registered future, when a nurse entered the house, to look for her name in the register, and see that she deserved her title. Now this last suggestion implies that a nurses' register is a thing that most people would keep about their houses, which Dr. Bedford Fenwick must know is very unlikely.

Let us take his favourite comparison—the medical register. Does Dr. Bedford Fenwick attend a single patient who has a medical register in his house, or would refer to it on sending for a doctor? Even if he did so, would he appreciate the difference between the M.D. of London and the L.K.Q.C.P., Dublin? And would he not prefer L.K.Q.C.P. to M.D. if the former cured him sooner than the latter? He would pay much less heed to length of scientific training than to practical results. It would be the same with the nurse. When there is illness in the house, those in charge do not go out of their way to purchase registers and enquire where a nurse has been trained. The doctor who recommends her, the institution from which she has come, are her best references; and both doctor and institution will value her more for a record of well-managed cases than for a course of training at the most approved school in the kingdom.

With regard to taking a nurse's name off the register for unprofessional conduct, we think the upholders of the registration scheme are strangely blind to present facts if they think that a nurse who is drunken or dishonest, or otherwise obviously unfit for her post, can long command remunerative employment. Dismissed from one institution, she may be taken on at another; but not for long. She will soon be found out and sent away; she will drop from the honourable ranks of the profession much more surely than if her name were merely erased from the list of nurses. She herself will blot it out.

"What becomes," asked a lady at the meeting in Wimpole-street, "of the medical men who are struck off the register? Do they continue to practise, and if so, can they be prosecuted for it?" They can be prosecuted for signing a death certificate and for some other things, but, as a matter of fact no one, as Dr. Bedford Fenwick admitted, takes the trouble to do it; and except from a social point of view the ostracism does not count for much. There is more than one doctor who has been struck off the register for advertising himself or some such cause, who makes an income which many of his registered brethren would be only to thankful for. It would be the same with the nurse. If her patients liked her they would send for her again and recommend her to their friends, whether she was registered or not. If she proved to be unpopular or incompetent, all the registration in the world would not earn her a living. Besides no Royal Charter ever confers penal or compulsory powers upon the society it incorporates. The British Nurses' Association, if it ever obtains a charter, will certainly not have power to strike a nurse's name off the register should one be established.

I have not touched now on the question of registration as a test of competence. It is unnecessary. The register can only certify that a nurse has devoted a certain amount of time to hospital work, and has then passed an examination; and the best authorities are agreed that examination is no real test of a nurse's fitness. But beyond that fact lies the other very practical one, that registration will in no degree enhance a nurse's market value.

### Notes from Australia.

*(By Our Own Correspondent.)*

Melbourne, April 25.

I ought, before this, to have sent you an account of the Melbourne Hospital, but, to tell the truth, I dread going there. It is a dreary-looking red-brick building, at the corner of two very busy streets. The matron is said to work excellently, but she is handicapped by the nurses, who begin by being pretty good charwomen, and by the crowded state of the wards, and the constant bickering about the new site. Melbourne has no more hospital accommodation now that it is a city of 420,000 inhabitants than it had when its population was 220,000, and naturally the pressure upon the accommodation is severe. At each fortnightly meeting the committee of the Melbourne Hospital calls public attention to the situation, and its hardships and cruelties, and endeavours to arouse the public from its apathy, and to relieve itself from responsibility. And this is right, for the subject is one which, for shame's sake, must speedily be grappled with. At a meeting held on the 12th with regard to this subject, the following two resolutions were passed: (1) "That this preliminary meeting, consisting of the Mayors of Melbourne and the surrounding suburbs, and the presidents and vice-president and members of the

Melbourne Hospital committee, is convinced of the necessity which exists for providing additional hospital accommodation for Melbourne and the surrounding suburbs." (2) "That a working committee, with power to add to their number, be appointed to draw up circulars and do other necessary work towards making the coming meeting a success, and to draft resolutions and arrange for proposers and seconders of the same."

About 100 beds in the Melbourne and 100 beds in the Alfred Hospital are occupied by typhoid cases, which partly accounts for the great pressure. When I next write I shall hope to inform you that there is a prospect of this shameful overcrowding being relieved.

Nurse Elizabeth Bradley, of the Melbourne Hospital, has been appointed head nurse at the Women's Hospital. This hospital is managed by a committee of women, though the men are doctors. Twenty in-patients a week, and 120 out-patients, and 20 midwifery cases, gives a nurse a fair scope for showing her powers, and it is to be hoped that Nurse Bradley will do well. I cannot believe that our nurses here are so well trained as they might be, and I wish more of our hospitals would import English matrons and sisters to reorganise the nursing.

The Premier has issued for publication the progress report of the Sanitary Commission. It is signed by Prof. H. B. Allen (chairman), Prof. Orme Masson, and others. The commission has met 39 times in Melbourne for the transaction of general business, and has examined 65 witnesses. Its members have inspected 11 slaughtering establishments and 66 noxious trades. In the pursuance of its investigations it has visited Sydney and Adelaide, where the local sewage systems were visited, and evidence taken upon sanitary matters generally. The commission has not concluded its investigation concerning the general sanitary condition of Melbourne and its suburbs, but it has collected sufficient evidence to lead it to condemn in the strongest language the present systems. When enquiring into the treatment of refuse matter, it found that the liquid refuse is conducted in the city in the first instance into the street channels.

The stagnant decomposing drainage also gives off offensive emanations which pollute the air. The underground sewers are not sufficiently ventilated, offensive gases escape through the various openings, and accumulations of a black and very offensive silt frequently occur, which is removed through manholes. During this process of removal the smell from the sewers is very offensive, and the air is necessarily polluted. The investigations conducted in Melbourne, Adelaide, and Sydney have convinced the commission that nothing short of a complete system of underground drainage will satisfy the requirements of Melbourne.

The commission adds that in certain suburbs, notably Footscray, Kensington, South Yarra, and Kew, the air is tainted by evil odours from noxious trades.

The inmates of Yarra Bend Lunatic Asylum had a picnic lately at which a newspaper correspondent was present, and he gives an amusing yet pathetic account of the proceedings. He says: "In the quadrille proper there are two or three merry fellows, attendants of the institution, who have blackened their faces and put on harlequin attire, and done much to make the day bright. It is almost funny to see them dancing with patients whose delusions are dignified. The whole party move on in stately style through the figures, bowing with a set grace and retiring with an evident impression that all is solemn earnest. And then when all is finished, just out on the skirts of the ring, there is an old Scotchman, who, as a protest, perhaps, against silly Southern ways, has chosen to 'dance his lane' in a Highland fling, and who flings away still, though the music has ceased."

The saying that "good springs out of evil" has not often been better illustrated than by the history of what is known to our kinsfolk at Melbourne as the Austin Hospital for Incurables. An incurable case was discharged from one of the hospitals. The big heart of a wealthy lady was greatly touched by the seeming cruelty of the proceeding. As soon as possible she devoted £6,000 towards providing a home specially for such cases; she got the Government and wealthy friends to help her, and now, on a hill at Heidelberg, about eight miles from Melbourne, stands the very admirable institution known as the Austin Hospital for Incurables. The situation is splendid, and the air perfect. In February of this year there were eighty-one patients, eleven of whom were suffering from cancer. One of the patients is at once blind and deaf and dumb, but instead of being the oppression of all about he has won the credit of being "the cleanest, nicest man in the house." He gives almost no trouble, and is always patient and always cheerful. There were other equally interesting cases, and an effort is to be made very speedily to build a fresh wing, to be devoted to the treatment of consumption. Victoria has sundry unquestionable advantages over the old country, and one of them is its more liberally proportioned care of the helpless and poor.

### Some New Wound Dressings.

SURGICAL dressings are to wounds what drugs are to the internal economy, but sometimes they are even more important. The most skilful manipulations of the surgeon are of small avail if, after his departure, the injured part is exposed to all kinds of dangerous poisons which may be floating in the air or be applied to the wound in the shape of injurious or unsuitable dressings. All great surgeons have attached the highest importance to the treatment succeeding their operations—the "after-treatment." It is, perhaps, quite safe to say that no man can be a great surgeon at all who does not enter with all his mind into the minutest details of wound dressing. What will make the patient comfortable; what will check or absorb poisonous discharges; what will promote the speedy healing of parts? These are questions which no conscientious and intelligent operator can afford for a moment to ignore.

Thanks to the thoroughly scientific and practical spirit of modern surgery, no part of the operator's work has received more careful attention than the details of "dressing." Sir Joseph Lister, who headed the movement which has revolutionised the surgeon s art, has devoted to every department of surgical work a minute and painstaking study which is beyond all praise. Other men of the highest eminence have followed in his footsteps, with the result that modern surgery has progressed by leaps and bounds; and at the present moment the world is full of its ever-increasing triumphs. In consequence of the marked advances in the knowledge and skill of surgeons, manufacturers of surgical appliances have all been put upon their mettle in order to keep pace with the demands of the exact and critical uses of their wares. A good many eminent firms have produced new dressings of varying degrees of excellence. Perhaps no single firm has placed upon the market so extensive a selection of articles of the first order of excellence as the Liverpool Lint Company. It must be borne in mind that excellence of material and adaptability for use are the two chief desiderata in surgical dressings, whilst the question of price is one of less importance. It is very seldom that manufactured articles of any kind can be both very inexpensive and very good. "Cheap and nasty" is an axiom well known to purchasers. We believe, however, that in price, as well as in quality, the articles furnished by the Liverpool Lint Company will compare favourably with those of any other firm. A brief description of some of the special productions of this company may prove both interesting and advantageous to hospital officials and other professional readers.

First among these may be noticed what is aptly called the "complete surgical dressing." It is so named because it is such a combination of materials as to constitute in itself a single and all-sufficient dressing for all ordinary wounds. What that means to the surgeon it is not necessary to explain at length. The "complete surgical dressing" is composed of very thick sheets of "absorbent" cotton wool. These sheets are for some purposes rendered antiseptic, usually by sublimate solution, and for others are, of course, plain. They are covered on the face with bleached gauze to prevent immediate contact of the fibres of the cotton wool with the raw surface of the wound. The backing consists of indiarubber of good quality. It is easy to see that by using this single "complete" dressing, instead of several distinct substances, a great saving of time is effected, and a much more comfortable and neat result is produced for the patient.

"Wound padding" is another speciality. It is somewhat similar in appearance to the "complete" dressing, but is made of a thick layer of carbolised tow incorporated with absorbent cotton wool. As in the "complete" dressing, it is faced with fine antiseptic gauze, and backed with impermeable backing. For all practical purposes it constitutes a complete dressing, as well as padding for wounds.

"Splint padding," as its name indicates, is used for all purposes where padding of any kind is required. It is made of carbolised tow and absorbent cotton wool, in thick, soft, and perfectly uniform layers. When first designed, it was with the object of supplying a long-felt want for a soft and entirely uniform material for the padding of splints. That object it still supplies, and its softness and uniformity in thickness render it all that is desirable. It is found, in practice, however, that not only is it perfect as padding for splints, but that, being antiseptic, it can, in many cases, supply the place of the complete dressing. Indeed, the practitioner whose cabinet is well supplied with the "complete" dressing, and the "wound padding" has practically all he is ever likely to require. For hospitals, of course, a wider range of selection is generally desirable. The splint padding, in addition to the almost universal use to which it can be put, has the further merit of being very inexpensive. When it is compared with the vast sheets of unprepared and uneven cotton wool, which formerly furnished for surgeons the only available "padding" they could find for splints, the advantages of the "splint-padding" are seen at a single glance.

"Proofed lint" is an article of a somewhat different class. It is used in the same way as ordinary lint for water dressings, and also for the application of ointments to wounds when such are required. Its great advantage lies in its perfect cleanliness, the "proofing" preventing the soiling of wearing apparel and bedclothes. In addition to its use by hospital surgeons and practitioners generally, the "proofed lint" will probably soon find its way into the family circle. It is one of those requisites which are in constant demand for cuts and other small injuries, and, as the advertisements say, "no family should be without it."

"Absorbent proofed felt" is a thick, soft, felted material with an impermeable backing, and is likely to come into extensive use both in the hospital and in the home for hot fomentations and other purposes where hot moist applications are required. It is made of varying degrees of absorbability, and, with its impermeable backing, is said to be not only clean and extensively useful, but superior to anything of the kind now in the market. It is, besides, very inexpensive, costing less than the ordinary spongios, whilst being more convenient and comfortable.

All these articles, samples of which have been submitted to us, are of undoubted excellence of material and workmanship, and cannot fail to be highly appreciated by scientific surgeons, whose purposes they will so admirably serve. Surgical dressings at the present day are both complicated and costly. These articles at least greatly reduce the degree of complication, and it is not improbable will, in the long run, be found more economical in money as well as in time than the ordinary dressings now in use. Hospital surgeons who have nurses and students to act as dressers may not find it essential to change their methods rapidly, even though the changes be demonstrable improvements. But private practitioners, to whom "time is money," may certainly be expected to adopt, without a day's delay, the new dressings, which will so greatly facilitate their labours. The merits of the articles are unquestionable, and are rapidly gaining approval. As one surgeon says, "Your complete dressings, etc., are quite the nicest things I have yet seen."

### Notice.

The Directress of the Hollond Institution, Nice, thanks all the nurses who have written to her, but as she cannot answer each one separately, she begs that those who do not receive letters from her *speedily* will not expect any reply.

### Everybody's Opinion.

[*Correspondence on all subjects is invited, but we cannot in any way be responsible for the opinions expressed by our correspondents. No communications can be entertained if the name and address of the correspondent is not given, or unless one side of the paper only be written on.*]

### NURSES AT NEWCASTLE.

Miss Balkville, matron, writes from the Royal Infirmary, Newcastle-on-Tyne: Perhaps a few words may give a fuller idea of our "Home of Rest" than the reference in your paper two weeks ago. The rooms have been chosen primarily for those nurses who do not live in the town, and who have no friends to invite them out to tea. The sitting-room has not only a couch, easy-chairs, and pleasant windows looking over an open park, but a cabinet containing teacups and saucers, and some fresh provision. The rooms are in a private house, and the good mistress sends to the baker's shop for some new cakes when her visitors come, and as her kettle is always boiling, tea is soon sent up to them. Books, games, etc., will soon be sent there for them. Already we find it is quite appreciated. A double-bedstead room also belongs, so that tired nurses may be sent two at a time, from 5 p.m. to 10 a.m., a plan which, I believe, will help to keep them in good health and spirits.

### Appointments.

[It is requested that successful candidates will send a copy of their applications and testimonials, with date of election, to The Editor, The Lodge, Porchester-square, W.]

Her Majesty's Hospital for Sick Children, Stepney Causeway, E.—Miss Annie Warburton was appointed matron of this institution on the 7th inst. It contains seventy beds, and the ages of the patients vary from babyhood to twenty years. Miss Warburton was trained at the London Hospital, her certificate being dated February 5, 1885. She also holds a diploma of the London Obstetrical Society. On the completion of her training, Miss Warburton was appointed staff-nurse at the City of Dublin Hospital, where she won golden opinions from everybody, which caused her to be selected for the position of lady superintendent to the Orthopædic Hospital, Dublin, at the end of 1886. In June, 1888, owing to illness, she resigned her appointment to the great regret of the hospital authorities, and was subsequently appointed acting lady superintendent of the Adelaide Hospital. Miss Warburton has the reputation of being an exceptionally good organiser, and we hope she will find her new sphere of work congenial in every way.

### Vacancies.

The following vacancies are announced:

**Lady Superintendent.**

Royal Victoria Hospital, Bournemouth, £50.

**Matron.**

Manchester Royal Eye Hospital, £80.

**Nurses.**

Bedford Institute, £26.
Blackheath Nursing Institution, £20.
Bradford Infirmary, £25.
Cardiff Infirmary.
Carmarthen Infirmary, £23.
Epsom Cottage Hospital, £18.
Harrogate Cottage Hospital.
Isle of Thanet Union (assistant), £25.
Johnson Hospital, Spalding, £20.
Lowestoft Hospital, £25.
Miss John's Institute, Glasgow.
Northampton Institution.
Nurses' Home, Harrogate.
Nurses' Institute, Bedford.
Nottingham Nursing Institution, £25.
Nurses' Home, Norwich.
Nursing Institution, St. Leonards.
Poplar and Stepney Asylum, £16 10s.
Potter's Bar Cottage Hospital, £30.
Princess Alice Memorial Hospital, £16.
Royal Hospital, Portsmouth, £25.
Southport Trained Nurses' Home.
St. Cuthbert's Poorhouse, £25.
St. George's Home, Sheffield.
Wantage Cottage Hospital, £25.
Workhouse Infirmary Nursing Association.

**District Nurses.**

Nurses' Home, Bolton, £25.

Bicester.

Penalverne, Penzance, £45.

Burton-on-Trent Institution, £25.

**Probationers.**

Burton-on-Trent Institution.
Bolton Infirmary
Birmingham Children's Hospital.
Coldstream Cottage Hospital.
Ventnor Consumptive Hospital, £10

